# Clinical implications of genome-wide DNA methylation studies in acute myeloid leukemia

**DOI:** 10.1186/s13045-017-0409-z

**Published:** 2017-02-02

**Authors:** Yan Li, Qingyu Xu, Na Lv, Lili Wang, Hongmei Zhao, Xiuli Wang, Jing Guo, Chongjian Chen, Yonghui Li, Li Yu

**Affiliations:** 1 Department of Hematology and BMT center, Chinese PLA General Hospital, 28 Fuxing Road, Beijing, 100853 China; 2 Department of Hematology, Hainan Branch of Chinese PLA General Hospital, Sanya, 572013 Hainan Province China; 3 Medical school of Nankai University, 94 Weijin Road, Tianjin, 300071 China; 4 Annoroad Gene Technology Co. Ltd, Beijing, 100176 China

**Keywords:** Acute myeloid leukemia, DNA methylation, Clinical implications

## Abstract

Acute myeloid leukemia (AML) is the most common type of acute leukemia in adults. AML is a heterogeneous malignancy characterized by distinct genetic and epigenetic abnormalities. Recent genome-wide DNA methylation studies have highlighted an important role of dysregulated methylation signature in AML from biological and clinical standpoint. In this review, we will outline the recent advances in the methylome study of AML and overview the impacts of DNA methylation on AML diagnosis, treatment, and prognosis.

## Background

Acute myeloid leukemia (AML) is characterized by clonal expansion of undifferentiated myeloid precursors, resulting in impaired hematopoiesis and bone marrow failure [1]. AML is a predominantly fatal hematopoietic malignancy with high heterogeneity [2–5]. Genetic heterogeneity has been appreciated in AML since early karyotyping studies [6]. With next-generation sequencing (NGS), genome studies of somatic mutations have shown a comprehensive landscape of AML and contributed to the understanding of the pathogenesis and progression of AML [5, 7–9]. A latest study of 1540 AML patients revealed distinct molecular subgroups that reflect discrete paths in the evolution of AML, informing disease classification and prognostic stratification [5]. It is well established that genetic aberrations play a critical role on the diagnosis, treatment, and prognosis of AML, which is fully reflected in the National Comprehensive Cancer Network (NCCN) guidelines for AML. However, nearly 50% of AML samples have a normal karyotype and many patients carry no mutation [10–12]. Meanwhile, DNA methylation patterns are altered in numerous cancers and often correlate with clinically relevant information such as subtypes, prognosis, and drug response [13–15]. Indeed, aberrant DNA methylation patterns are a hallmark of AML [16–18]. Despite the recognized relationship between DNA methylation and AML, the development of methylome assessment is limited by the lack of rapid, reliable assays that provide validated information. Recently, the advance of technologies, e.g., DNA methylation microarrays and next-generation sequencing [19–25], has made methylome analysis less time-consuming, reproducible, and cost-effective [24, 26], and the genome-wide coverage has been extended to non-CpG island regions, e.g., enhancer, exon, intron, and intergenic [21, 24, 25, 27]. With high accuracy and robustness, DNA methylation analysis has been confirmed to be feasible and reliable in clinical diagnosis and precision medicine, especially for highly heterogeneous diseases such as AML [26, 28, 29]. There are now an increasing number of studies reporting aberrant DNA methylation in AML [30–34], and new methods for detecting DNA methylation on a genome-wide scale have significantly widened our knowledge about aberrant methylation patterns in AML. For example, distinct DNA methylation patterns are used to define AML subgroups and a set of aberrantly methylated genes are identified and linked to the clinical outcome [9, 30, 35]. Additionally, DNA methylation and mutation patterns may occur with distinct kinetics to affect the biological and clinical features of AML [9].

### Distinct DNA methylation patterns identified in AML

**Fig. 1:**
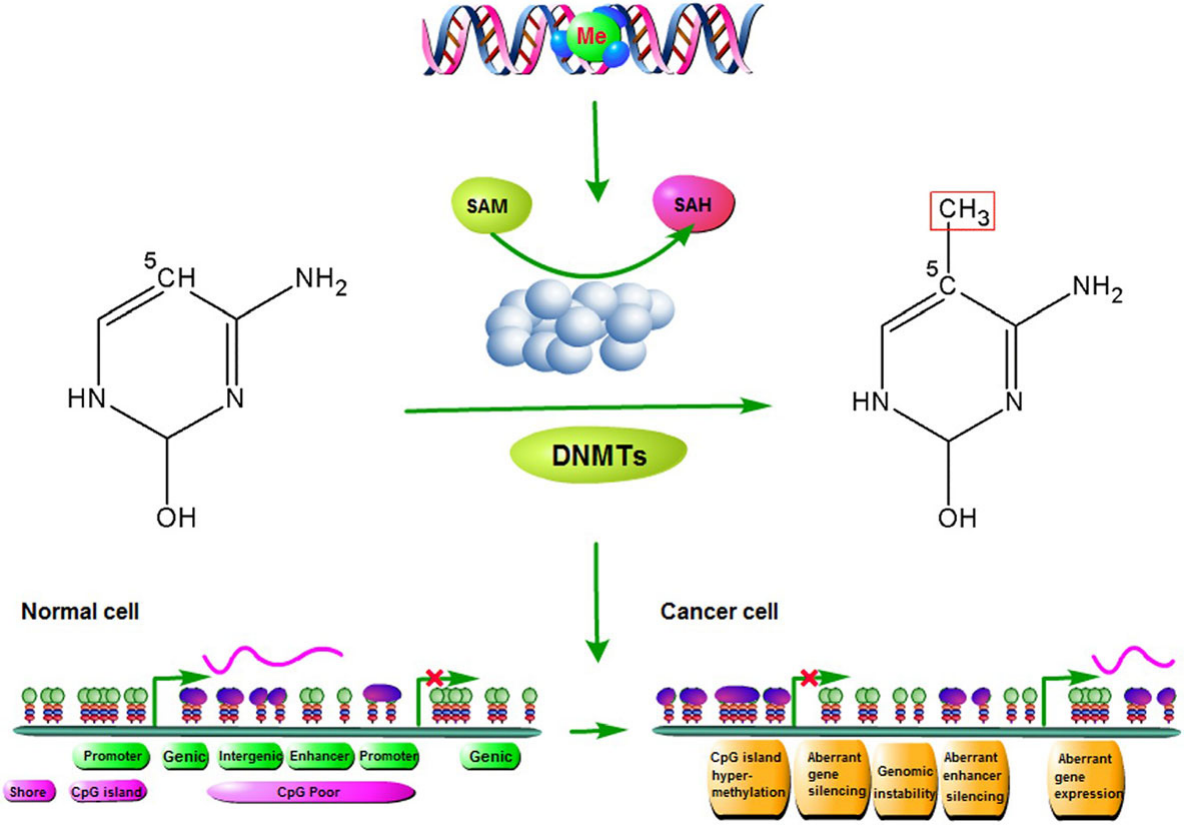
DNA methylation and deregulation of the genome in carcinogenesis. Methylation of cytosine within CpG dinucleotides is catalyzed by DNMTs. S-adenosylmethionine (SAM) donates methyl groups and is converted to S-adenosylhomocysteine (SAH). In normal cells (*lower left*), CpG islands are often associated with gene promoters and are resistant to DNA methylation. Gene expression can occur and is highly correlated with high levels of gene body (genic) methylation. CpG-poor regions (intergenic), except for enhancers, are typically methylated, while CpG-poor promoters are silenced by DNA methylation unless gene expression is required in specific tissue. In cancer cells (*lower right*), CpG islands are prone to DNA hypermethylation, which results in aberrant gene silencing (e.g., of tumor suppressor genes). Concomitant hypomethylation of intergenic regions and CpG-poor promoters contributes to genomic instability and aberrant gene expression (e.g., of oncogenes), respectively. *Green circle*, unmethylated CpG; *purple circle*, methylated CpG

AML is a highly heterogeneous disease with fewer mutations than most other adult cancers [7]. This difference suggests that other mechanisms, e.g., epigenetics or post-transcriptional regulations to play a pivotal role in determining the biological behavior of the disease. DNA methylation is the major mode of epigenetic modification [36–38], which plays an important role in carcinogenesis (Fig. 1). Aberrant DNA methylation patterns are a characteristic feature of AML [7, 17, 18]. Several studies have evaluated genome-wide methylation in AML [7, 9, 30, 39]. The Cancer Genome Atlas Research Network (TCGA) performed methylation profiling for 192 samples of AML using Illumina Infinium HumanMethylation450 BeadChip and identified significant changes in DNA methylation at 160,519 CpG loci, which accounted for 42% of sites tested, with 67% resulting in hypermethylation and 33% resulting in hypomethylation [7]. A pairwise AML cohort study examining the DNA methylation by enhanced reduced representation bisulfite sequencing (ERBBS) based on NGS platform suggested that global DNA methylation allele shifting was a universal feature of AML relative to normal bone marrow controls [9]. Another detailed study on the genomic DNA methylation landscape profiling using HpaII tiny fragment enrichment by ligation-mediated PCR (HELP) methylation microarrays revealed the existence of 16 distinct DNA methylation patterns in AML [30]. Each of these DNA methylation-defined AML subtypes displayed a unique epigenetic signature when compared with the normal bone marrow CD34+ cells. Though 11 of 16 clusters correspond to AML subtypes defined by the World Health Organization (WHO) or related to specific genetic and epigenetic lesions, 5 new clusters could not be explained based on known morphologic, cytogenetic, or molecular features. In fact, each of these AML subtypes displays a distinct DNA methylation pattern. Although this scenario was previously proposed, the findings represent an important progress made possible by the use of large-scale genome-wide DNA methylation profiling technology.

Cytogenetically normal AML (CN-AML), which constitutes between 40 to 50% of all AML cases [40], is the most heterogeneous group in AML. Interestingly, a genome-wide differential methylation study in CN-AML using Illumina 450 K methylation array found that the most pronounced changes in DNA methylation occurred in non-CpG island regions, whereas hypermethylation enrichment was only represented in CpG islands [41].

It is foreseeable that future research will provide more clarity and precision to the methylome landscape of AML.

### DNA methylation in diagnosis classification of AML

Recent genome-wide studies identified DNA methylation signatures unique for subtypes of AML patients [30], which could be valuable for diagnosis classification of AML [7, 9, 30]. Li et al. [9] examined the epigenetic heterogeneity by ERRBS in serial diagnosis-relapse pairwise AML samples and defined three categories of DNA methylation-shifted loci: loci unique to diagnosis, loci unique to relapse, or loci present at both diagnosis and relapse. This analysis segregated AML patients into three clusters with no significant association with age, white blood cell count (WBC), or the French–American–British (FAB) classification, suggesting that DNA methylation pattern could be an independent diagnosis classification for AML patients. Furthermore, different cytogenetic and molecular subtypes were found to exhibit highly distinct DNA methylation profiles [7, 17, 30, 39, 42], providing a new perspective for diagnosis classification of AML. For cases with t(8;21), inv(16) or t(16;16), t(15;17) or t(v;11q23) translocations, or the presence of the relevant fusion genes, unique DNA methylation signatures can define these AML subtypes [7, 17, 30, 39]. Accentuated DNA hyper- and hypomethylation were both identified in t(8;21)-*AML1/ETO* and inv(16)-t(16;16)-*CBFB-MYH11* by Illumina 450 K, with hypomethylation being the predominant feature. However, almost equally accentuated DNA hyper- and hypomethylation was found in t(15,17)-*PML-RARa*. Unlike these DNA methylation patterns, a very pronounced DNA hypomethylation signature was found in t(v;11q23)-*MLL* translocations [7, 30, 33]. It is proposed that the underlying mechanism of aberrant DNA methylation induction in these AML was that these fusion genes might recruit DNA methyltransferases (DNMTs) to their binding site [43–45]. In addition, secondary epigenetic dysregulation might also contribute to the aberrant methylation, which includes the binding of PML-RARa to genomic regions of epigenetic modifiers such as *DNMT3A* and/or DNA methylation disruption of AML1-ETO target genes [46–48]. In a recent study of 60 acute promyelocytic leukemia (APL) primary samples at diagnosis, methylation of *DAPK1*, *miR-34a* and *-34b/c* were tumor-specific in APL [49]. Hájková et al. reported a novel hypomethylation pattern specific to *CBFB-MYH11* fusion resulting from inv(16) rearrangement using targeted bisulfite sequencing in AML patients [42]. They found that average levels of DNA methylation in assigned regulatory regions of *MN1*, *SPARC*, *ST18*, and *DHRS3* were significantly lower for inv(16) compared to non-inv(16) AML M4, other AML subtypes, and healthy controls (*p* < 0.0001).

Apart from translocations or the relevant fusion genes, recurrent mutations (e.g., *NPM1*, *CEBPA*, *RUNX1*) in AML can be defined by DNA methylation differences, especially for mutations in epigenetic regulator genes (e.g., *DNMT3A*, *TET2*, *IDH1/2*) [7, 30, 42]. For *NPM1* mutations, four DNA methylation clusters were identified: one hypermethylated and three both hyper- and hypomethylated identified using HELP [30], the strong hypomethylation signature identified using Illumia 450 K [7], and the hypermethylation signature identified using MethylCap-seq [50]. For *CEBPA* double mutations, the cases could be split to two distinct subtypes with different methylation signatures: one hypermethylated and one hypomethylated identified using HELP [30], and the DNA hypermethylated signature identified using Illumina 450 K [7]. However, discrete hyper- and hypomethylation signatures were showed for *RUNX1* mutations using Illumina 450 K [7].

*DNMTs* (*DNMT1*, *DNMT3A*, and *DNMT3B*) encode methyltransferases that catalyze the addition of a methyl group to the cytosine residue of CpG dinucleotide to maintain methylation status of hematopoietic stem and progenitor cells [51, 52]. *DNMT3A* is the essential DNA methylation regulator, was thought to have a severe impact on DNA methylation patterns [53, 54]. Mutations in *DNMT3A* contribute to dysregulation of DNA methylation may result in global shifts in gene expression in hematologic malignancies, which frequently leads to increased self-renewal in blood cells at the expense of normal differentiation [51, 55, 56]. *DNMT3A* mutations are present in preleukemic hematopoietic stem cells (HSCs), and it is considered an early event in AML [57]. Qu et al. demonstrated that *DNMT3A* mutations were a main genetic contributor to the global methylation pattern, and two CN-AML subtypes were generated according to the samples with or without *DNMT3A* mutations [41]. Additionally, Marcucci et al. noted that only *DNMT3A-R882* mutations were associated with hypermethylation [50]. Furthermore, *TET2* and *IDH1/2* mutations resulted in genome-wide DNA hypermethylation signature, especially for *IDH1/2* mutations [7, 16, 39, 50]. A meta-analysis also supported the diagnostic value of DNA methylation in leukemia with 41 case-control studies [58]. In this study, 20 genes were found to be aberrantly methylated in the leukemia patients, and *CDKN2A*, *CDKN2B*, and *ID4* genes were significantly hypermethylated in AML. Though recent studies have identified the relationship between DNA methylation abnormalities and AML variability [17, 30, 39], more details remain to be revealed and many mechanisms remain unclear [17, 59]. Nevertheless, the value of DNA methylation in the diagnosis stratification of AML cannot be underappreciated.

### DNA methylation in prognostic stratification of AML

Many studies have found that DNA methylation could predict clinical outcome in AML patients and aberrant DNA methylation can serve as a biomarker for risk stratification (Table 1) [9, 16, 31, 33–35]. However, the results were inconsistent due to the difference in AML cohort, genomic regions analyzed, functions of annotated methylated genes, and methods of detection and analysis. Deneberg et al. [31] reported that global and gene-specific methylation patterns were independently associated with the clinical outcome in AML patients. They analyzed the methylation of *CDKN2B*, *E-cadherin (CDH)* and *hypermethylated in cancer 1 (HIC1)* promoters, and global DNA methylation in 107 AML patients by the luminometric methylation assay (LUMA). They also assessed genome-wide promoter associated methylation using the Illumina HumanMethylation27 array in 20 patients. Multivariate analysis suggested that low global DNA methylation was associated with higher complete response (CR) rate, and increased genome-wide promoter associated methylation was associated with better overall survival (OS) and disease-free survival (DFS). Furthermore, *P15* methylation was associated with better OS and PFS, while *CDH* and *HIC1* methylation was not associated with clinical outcome [31].

**Table 1:** Prognostic genes regulated by DNA methylation identified in AML by genome-wide, large sample studies

Reference	DNA methylation detection methods	AML group	Prognostic genes regulated by DNA methylation
Figueroa et al. [16]	HELP	344 Newly diagnosed AML	*BLR1 (CXCR5)*, *BTBD3*, *E2F1*, *FAM110A*, *FAM30A*, *GALNT5*, *KIAA1305*, *LCK*, *LMCD1*, *PRMT7*, *SLC7A6OS*, *SMG6*, *SRR*, *USP50*, *VWF*, *ZFP161*
Li et al. [9]	ERRBS	138 Paried AML (diagnosis and relapse)	*CCDC85C*, *CHL1*, *ELAVL2*, *FAM115A*, *FAM196A*, *GPR146*, *GPR6*, *HELZ2*, *ID4*, *IL2RA*, *KCNG3*, *LOC254559*, *LOC284801*, *NPAS2*, *PCDHAC2*, *PROB1*, *SHISA6*, *SLC18A3*, *SOCS2*, *TRIM67*, *ZFP42*
Marcucci et al. [50]	MethylCap-seq	134 CN-AML (355 CN-AML validated)	*AATK*, *ACAP3*, *ADCK2*, *ADCY6*, *AGPAT9*, *AHCY*, *ALOX15B*, *ANXA6*, *APBB1*, *APOD*, *AQP11*, *ARHGAP27*, *AXL*, *BRF1*, *C15orf62*, *C17orf77*, *C8orf51*, *CABLES1*, *CARD11*, *CD34* ^*a*^, *CHMP7*, *CISH*, *CLDN15*, *CLEC3B*, *DDIT4*, *DHCR24*, *DHRS12*, *EGFL7*, *ETS1*, *EVC*, *F2RL1* ^*a*^, *FAM92A1* ^*a*^, *FCHO1*, *FKBP4*, *FLVCR1*, *FLVCR1-AS1*, *FZD6*, *GAL3ST3*, *GCNT2*, *GIT1*, *GPR56*, *H1F0*, *HCN2*, *HIVEP3*, *IQSEC1*, *KCNK6*, *KDM2B*, *KLHL3*, *KNCN*, *LOC646627*, *MDFI*, *ME3*, *MEOX1*, *MIR126*, *MIR155HG* ^*a*^, *MVD*, *NAV1*, *NBL1*, *NLRP1*, *PLK3*, *PMM1*, *PRKCZ*, *PRKG2*, *RAB36*, *RGS3*, *RHOC* ^*a*^, *RHPN1*, *SCARF1*, *SCRN1* ^*a*^, *SH3TC1*, *SPRY1*, *SRC*, *TBL2*, *TCEA3*, *TENC1*, *UBXN6*, *VWA8* ^*a*^, *WDR16*, *WDR86*, *WRAP53*, *ZNF623*, *ZNF70*

^*a*^Seven genes (*CD34*, *RHOC*, *SCRN1*, *F2RL1*, *FAM92A1*, *MIR155HG*, and *VWA8*) had not only DNA methylation regions (DMRs) but also expression levels that were associated with outcome

Figueroa et al. analyzed distinct DNA methylation signatures, identified new AML subtypes, and explored the potential use of aberrant DNA methylation as a predictor of important clinical features. With a three-step approach of model development and validation using a large data set, they reported a 15-gene methylation classifier predictive of OS [30]. These results suggested that DNA methylation classifier could serve as a clinically useful biomarker. Luskin et al. [35] recently reported a validated clinical measure of DNA methylation, *M* score, generated from expedited HpaII small fragment enrichment by ligation-mediated PCR (xMELP) assays [60, 61] that represent a binary prognostic classifier for patients with de novo AML. The *M* score was robustly associated with CR and OS in both univariable and multivariable models in multiple independent AML cohorts, as well as for AML patients aged ≤60 years with intermediate cytogenetics [35]. A high *M* score represented a shorter 2-year OS (24 vs 56%) and a lower CR rate (61 vs 84%) compared with a low *M* score. These findings confirmed the association of *M* score with clinical outcome, which has been further validated in an independent cohort of patients with APL and secondary AML [62]. Remarkably, the association of *M* score with clinical outcome was stronger than that of many established prognostic factors, including cytogenetics, *FLT3-ITD* status, and other genetic lesions. Additionally, the *M* score classifier also defined subgroups with significantly OS within a traditionally high-risk subgroup with intermediate cytogenetics and *FLT3-ITD* mutation. These results suggest that DNA methylation can be used for risk stratification, which might decrease the need for comprehensive genetic testing for risk stratification at diagnosis due to its better prognostic performance [35].

Similarly, a recent NGS study pointed out that epigenetic and genetic heterogeneity occurred with distinct kinetics in AML. The changes in DNA methylation burden were independent of the abundance of somatic mutations in patients, and relapsed AMLs showed variable changes in DNA methylation burden, which was antecedent to the genetic evolution. Furthermore, the variance of CpG methylation patterns (measured as EPM) were associated with the time to relapse, whereas the burden of somatic mutations was not. The patients with high EPM at diagnosis had a shorter time to relapse compared to the low-EPM cohort (*p* = 0.0396), which was most significant for EPM values assessed from promoter-annotated epigenetically shifted loci (*p* = 0.0077) [9]. The study also detected a specific set of 21 promoter-annotated DNA methylation shifted loci to be associated with a shorter time to relapse, which could be used as outcome biomarkers [9]. Using MethylCap-seq, Marcucci et al. identified 82 individual genes, the promoter different methylation regions (DMRs) of which were associated with OS in a set of older patients with CN-AML [50]. For 80 genes, higher DMR methylation was related to longer OS. Combined with the expression data, a novel seven-gene score for clinical prognosis was generated validated in four independent CN-AML patient sets (*n* = 355). In multivariable analyses, patients with low scores had a more than 80% increase in the odds of achieving CR and approximately 3.5-fold decrease in the risk of disease relapse or death compared with patients with high scores [50]. Using targeted bisulfite sequencing, Hájková et al. [42] revealed that *PBX3* differential methylation could impact on prognosis of AML. They found that the hypomethylation of *PBX3* regulatory region was involved in higher relapse rates and shorter relapse-free survival in AML patients with overexpressed *PBX3*. However, this methylation signature was not related to OS.

### DNA methylation in therapeutic decision-making of AML

Variable responses to chemotherapy in AML represent a major treatment challenge, and the ability to predict therapeutic response is essential for improving the care of patients with AML. However, clinical and genetic features incompletely predict outcome, especially for CN-AML and AML with no mutation [10–12]. In general, DNA methylation might only be able to predict the response of hypomethylating agents [63–65]. For example, in a study investigating the impact of global and gene-specific DNA methylation status (promoters of 5 stem cell-related transcription factor genes *SOX2*, *OCT4*, *KLF4*, MYC, and *NANO*) in AML patients treated with decitabine [65], Zhang et al. showed that patients with a high level of 5-mC had a poor prognosis after demethylation therapy, and higher methylation status of the *SOX2* and *OCT4* genes was associated with differential response to demethylation therapy. This study found that relatively low methylation percentage in one or both of these two genes was also associated with longer OS after decitabine-based chemotherapy.

In fact, due to the complex epigenetic regulation mechanisms in AML, DNA methylation contributed to the overall biological and clinical features of AML and was also correlated with conventional chemotherapy [35, 66]. A decade ago, Grövdal et al. showed a significant effect of the methylation status of three genes (*P15ink4b (P15)*, *E-cadherin (CDH)*, and *hypermethylated in cancer1 (HIC)*) on the outcome of conventional chemotherapy using bisulfite-denaturing gradient gel electrophoresis (DGGE) [66]. Luskin et al., as mentioned previously, also assessed the impact of high-dose (90 mg/m^2^) or standard-dose (45 mg/m^2^) daunorubicin induction chemotherapy on a cohort AML patients by dividing the patients into low and high *M* score subgroups. They found that high-dose daunorubicin (90 mg/m^2^) was beneficial for patients with high *M* scores but not for those with low *M* scores. The different responses suggested that *M* score may be correlated with chemoresistance and could be used for identifying patients that might benefit from high-dose chemotherapy, which will contribute to therapeutic decision-making of AML [35].

In addition, the mutations in genes involved in DNA methylation (e.g., *DNMT3A*, *IDH1/2*, *TET2*) play an important role in genome-wide methylation signature in AML and contribute to the leukemogenesis and prognosis [16, 53, 67–69]. The applications of DNMTs and *IDH1/2* inhibitors have been more extensive and improved the outcome of AML via reversing abnormal DNA methylation and restoring normal hematopoiesis [52, 56, 70]. Two DNMTs inhibitors, azacitidine and decitabine, have been approved for MDS and AML due to the increasing data to support the efficacy of these hypomethylating agents (HMAs) [71–76]. Especially, the particular gene mutations, such as those in *DNMT3A* and *TET2* and methylation signatures, may predict for responsiveness to treatment with HMAs according to the studies in MDS [27, 77]. *TET2* mutations and/or *DNMT3A* mutations were independent predictors of better response (*p* = 0.03) and improved PFS (*p* = 0.04) [77]. While a 21 selected tile regions revealing the DNA methylation differences can served as an epigenetic classifier that accurately predicted decitabine response at the time of diagnosis [27]. Following this line, it is possible that defined AML subtypes with certain changes associated DNA methylation are more responsive to HMAs than others. With DNA methylation profiling identified in AML subgroups and the evaluation of DNA methylation level with clinical outcome, extending the methylome analysis to comparable studies is of great interest as these results would have immediate implications for design of therapeutic regimens, especially dissect which AML subtypes may benefit from treatment with HMAs [16, 35, 62].

Similar to *DNMT3A* and *TET2* mutations, *IDH1/2* mutations also could predict a favorable response with a significantly higher clinical remission rate during treatment with HMAs, and the odds of achieving response with an *IDH* mutation was 14.2 when compared to patients without an *IDH* mutation (95%CI, 1.3–150.4) [78]. Furthermore, hypermethylated signature in AML with *IDH* mutations could be reversed via *IDH* inhibition [16, 79]. *IDH1/2* inhibitors (e.g., IDH305, AG-220, AG-221) have been developed and are already being evaluated in clinical trials (Table 2) [70, 80]. Primary results suggest a prominent effect of these drugs in AML prognosis [81–85]. AG-120, an oral, first-in-class *IDH1* inhibitor, has shown the efficacy and safety with determined *IDH1* clearance as a single agent in patients with *IDH1*-mutant hematologic malignancies. The overall response rate (ORR) was 38.5% (30/78) [84]. A phase I study with IDH305 including 21 relapsed/refractory AML subjects enrolled reported similar results that 7 (33%) patients obtained objective responses with a favorable safety profile [83]. AG-221 is an oral first in class inhibitor of the *IDH2-*mutant protein. Preliminary results of a phase 1/2 study enrolled relapsed/refractory AML patients showed that AG-221 was well-tolerated and -induced responses in heavily pretreated RR-AML. Of the 138 enrolled AML patients, 128 were evaluated for efficacy and the ORR was 41% (52/128) [86]. Therefore, identification of mutations associated DNA methylation and evaluation the change of methylation signature would contribute to individual therapy of AML.

**Table 2:** Clinical trials with compounds of IDH inhibitors in patients with hematologic malignancies

Compound	Target	Phase	Registration number	Reference
IDH305	IDH1	1	NCT02381886	83
AG120	IDH1	1	NCT02074839	84
AG120	IDH1	1	NCT02073994	82
AG221	IDH2	1/2	NCT01915498	86
AG221	IDH2	1/2	NCT02273739	NA
AG221	IDH2	3	NCT02577406	NA
AG-120/AG-221	IDH1/IDH2	1	NCT02632708	NA
AG-120/AG-221	IDH1/IDH2	1b/2	NCT02677922	NA

*NA* no data about reference

### DNA methylation, genetic aberrations, and expression in AML

Genetic lesions and epigenetic abnormalities have been shown to play important roles in AML. Although the relationship of DNA methylation, genetic aberrations, and expression is unclear, it is likely that these parameters are closely related with each other [7, 30, 64, 87–89]. The TCGA study generated a genomic and epigenomic landscapes of AML, which would serve as a foundation for investigations AML pathogenesis, classification, and risk stratification [7]. A recent study by Papaemmanuil et al. identified 5234 driver mutations across 76 genes or genomic regions in 1540 patients with AML. The mutations in genes that encode DNA methylation regulators (e.g., *DNMT3A*, *IDH1/2*, *TET2*) were often acquired the earliest and with a high recurrence rate. Particularly, 73% of the largest class in their cohort, *NPM1*-mutated AML, also carried mutations in DNA methylation genes (*DNMT3A*, *IDH1*, *IDH2R140*, and *TET2*). Besides, they identified a subgroup of AML with *IDH2R172* mutations [5]. Since these mutations resulted in abnormalities of genome-wide DNA methylation signature, the relationship between genetic aberrations and DNA methylation were inseparable in AML [18, 50, 89]. Furthermore, Taskesen et al. created the three different classification strategies based on gene expression and DNA methylation profiles (GEP and DMP) from 344 well-characterized AML samples [87]. They demonstrated that prediction of known cytogenetic and molecular abnormalities in AML could be further improved by integrating GEP and DMP profiles. Raj et al. also provided insight into the clinical relevance of prognostic mutations and the mutation-associated gene DNA methylation promoter and expression patterns [63]. In a mouse model that has a defined leukemia stem cell population with a characteristic transcriptional and epigenetic profile, it was confirmed that *TET2* and *FLT3* mutations cooperated to induce AML, and the methylation changes exhibit the cooperation of disease alleles to target multiple loci. The data also suggested that leukemic transformation by these epigenetic changes is reversible and therapies that reactivate silenced genes might improve outcomes for AML patients [88].

## Conclusions

**Fig. 2:**
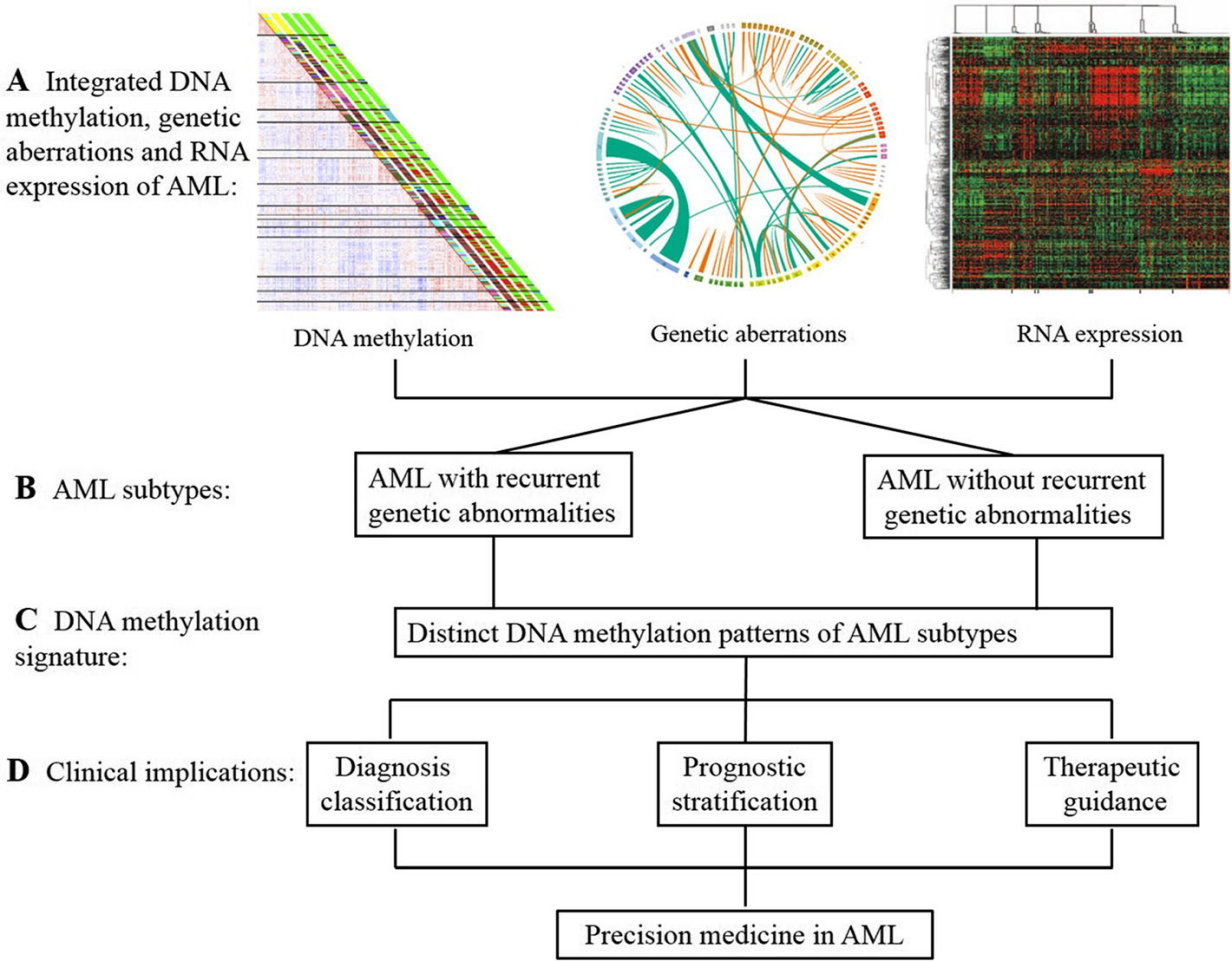
Paradigm of integrated DNA methylation, genetic aberrations, and expression of AML leading to precise medicine. The integrated analysis of DNA methylation, genetic aberrations (gene fusions and mutations), and RNA expression (**a**) has revealed multiple AML subtypes, summarized into two groups (with or without recurrent genetic abnormalities) (**b**). Furthermore, each subtype will be characterized with distinct DNA methylation patterns (**c**), which play an important role in clinical implications (**d**), leading to precision medicine in AML. The clinical implications of DNA methylation are discussed more detail in the text

DNA methylation is a common theme in acute myelogenous leukemogenesis. With the progress of technologies in identifying DNA methylation [24–26, 28, 29], especially the milestones in data integration, sharing, and analysis strategies, such as the International Human Epigenome Consortium Data Portal (IHEC Data Portal) [90], the BLUEPRINT Data Analysis Portal (BDAP) [91], and the tool for identifying cell type-specific signal in epigenomic data (eFORGE) [92], DNA methylation will be more widely used in clinical practice and become more valuable in diagnosis classification, prognostic stratification, and therapeutic decision-making of AML. This will contribute to the development of precision medicine in AML. Besides, a further understanding of the relationship among DNA methylation, genetic aberrations, and expression might provide unprecedented insights into the pathogenesis of AML (Fig. 2).

## Abbreviations

5-mC: 5-Methylcytosine
AML: Acute myeloid leukemia
AML1/ETO: AML1 and eight twenty-one rearrangements
APL: Acute promyelocytic leukemia
BDAP: The BLUEPRINT Data Analysis Portal
CBFB-MYH11: Core-binding factor beta and myosin heavy chain 11 rearrangements
CDH: E-cadherin
CDKN2A/2B: Cyclin dependent kinase inhibitor 2A/2B
CEBPA: CCAAT/enhancer binding protein alpha
CN-AML: Cytogenetically normal AML
CR: Complete response
DFS: Disease-free survival
DGGE: Bisulfite-denaturing gradient gel electrophoresis
DHRS3: Dehydrogenase/reductase 3
DMP: DNA methylation profiles
DMR: Different methylation region
DNA: Deoxyribonucleic acid
DNMT3A: DNMT 3 alpha
DNMTs: DNA methyltransferases
eFORGE: The tool for identifying cell type-specific signal in epigenomic data
EPM: The global metric eloci per million loci
ERBBS: Enhanced reduced representation bisulfite sequencing
FAB: French–American–British
FLT3-ITD: Fms-related tyrosine kinase 3 internal tandem duplication
GEP: Gene expression profiles
HELP: HpaII tiny fragment enrichment by ligation-mediated PCR
HIC1: Hypermethylated in cancer 1
HMAs: Hypomethylating agents
HSCs: Hematopoietic stem cells
ID4: Inhibitor of DNA binding 4
IDH1/2: Isocitrate dehydrogenase (NADP(+)) 1/2
IHEC: Data Portal the International Human Epigenome Consortium Data Portal
Klf4: Kruppel-like factor 4
LUMA: Luminometric methylation assay
MLL: Mixed lineage leukemia
MN1: MN1 proto-oncogene, transcriptional regulator
MYC: v-Myc avian myelocytomatosis viral oncogene homolog
NCCN: National Comprehensive Cancer Network
NGS: Next-generation sequencing
NPM1: Nucleophosmin
OCT4: Organic cation/carnitine transporter4
OS: Overall survival
PBX3: PBX homeobox 3
PML-RARa: Promyelocytic leukemia and retinoic acid receptor alpha rearrangements
RUNX1: Runt related transcription factor 1
SOX2: SRY-box 2
SPARC: Secreted protein acidic and cysteine rich
ST18: ST18, C2H2C-type zinc finger
TCGA: The Cancer Genome Atlas Research Network
TET2: Tet methylcytosine dioxygenase 2
WBC: White blood cell
WHO: World Health Organization
xMELP: Expedited HpaII small fragment enrichment by ligation-mediated PCR
